# The Human Milk Oligosaccharide Lacto-N-Fucopentaose III Conjugated to Dextran Inhibits HIV Replication in Primary Human Macrophages

**DOI:** 10.3390/nu17050890

**Published:** 2025-03-02

**Authors:** Tablow Shwan Media, Medhini Ramesh, Olivia Isa Lee, Lucy Njideka Ubaka, Donald A. Harn, Thomas Norberg, Frederick Quinn, Ankita Garg

**Affiliations:** 1 Department of Infectious Diseases, College of Veterinary Medicine, University of Georgia, Athens, GA 30602, USA; tablow01@gmail.com (T.S.M.); medhini.ramesh@uga.edu (M.R.); olivialee@uga.edu (O.I.L.); lucy.ubaka@uga.edu (L.N.U.); dharn@uga.edu (D.A.H.); fquinn@uga.edu (F.Q.); 2 Department of Biochemistry-BMC, Uppsala University, 753 10 Uppsala, Sweden; thomas.norberg@kemi.uu.se

**Keywords:** HIV-1, LNFPIII, β-chemokines, Gagp24

## Abstract

**Background/Objectives**: Individuals with HIV on combined antiretroviral therapy (ART) with virologic suppression exhibit chronic immune activation and immune dysfunction. Numerous studies have shown that human milk oligosaccharide (HMO) controls the postnatal transmission of HIV-1, but its effect on adult HIV-1 infection is not known. The purpose of this study was to investigate the anti-HIV activity of Lacto-N-fucopentaose III (LNFPIII) in adult blood-borne macrophages. **Methods**: Primary human monocyte-derived macrophages from the blood of HIV-seronegative individuals were infected with HIV and treated with or without dextran-conjugated LNFPIII (P3DEX). HIV replication was measured by quantifying the accumulation of HIV Gag p24 in the culture supernatants by ELISA. The quantities of chemokines MIP-1α, MIP-1β, and CCL5 in the culture supernatant were also measured by ELISA. The expression of IL-1β, IL-18, TNFα, IL-10, BECN1, and housekeeping gene HuPO in the macrophages was determined by qRT PCR. The expression of NF-kB, LC3, p62, and β-actin was measured by immunoblotting. **Results**: We found that P3DEX controls HIV replication without affecting HIV binding and/or internalization by human macrophages. The treatment of HIV-infected macrophages with P3DEX increased the quantity of beta (β)-chemokines MIP-1α, CCL5, and MIP-1β, which are known to have anti-HIV activity. Furthermore, the treatment of HIV-infected macrophages with P3DEX increased autophagic flux in a TLR8-dependent manner and ameliorated the expression of proinflammatory cytokines. These results suggest that P3DEX is a prominent milk-derived sugar that simultaneously augments anti-viral mechanisms and controls immune activation. These findings prudently justify the use and clinical development of P3DEX as a host-directed therapeutic option for people living with HIV.

## 1. Introduction

Human immunodeficiency virus 1 (HIV-1) infection causes profound immune suppression, leading to the progressive destruction of the immune system, the development of acquired immunodeficiency syndrome (AIDS), and, ultimately, death of untreated patients. In 2021, there were 38.4 million people living with HIV globally, with 1.5 million new cases of HIV infection reported, illustrating that HIV is still a very prevalent illness which requires attention [1]. While combined antiretroviral therapy has substantially reduced mortality and morbidity, chronic immune activation contributes to non-AIDS morbidity and all-cause morbidity in people living with HIV [2,3,4,5,6,7,8,9]. There is a global effort toward the development of host-directed therapies that could control immune activation. Several potential interventions, such as statins, probiotics, chloroquine, methotrexate, and ribavirin, have been looked at to control immune activation in people living with HIV (PWH). These treatments have shown inconsistent outcomes in their ability to control inflammation, and some even increased HIV titer in infected individuals [10,11,12,13,14,15,16,17,18]. Only antiretroviral intensification with raltegravir and maraviroc reduced immune activation and coincident control of HIV replication [19,20,21,22,23]. However, similar to other biologic and pharmacological agents, these demonstrated inconsistent outcomes in observational and randomized control trials [21,23,24,25,26,27]. Macrophages are the first cells to interact with and be infected by HIV. Notably, in individuals with HIV with depleted CD4+ T cells, HIV infection becomes increasingly macrophage-tropic. In these individuals, macrophages become the primary host cells with actively replicating virus and long-lived HIV reservoirs [28,29,30,31,32,33,34]. Thus, there remains a need to identify adjunctive host-directed therapeutic agents that can simultaneously augment anti-viral mechanisms and control immune activation in HIV-infected macrophages.

Human milk oligosaccharides (HMOs) are the third most abundant component of breast milk and control postnatal HIV transmission in a concentration-dependent manner [35,36,37,38]. HMOs are highly complex oligosaccharides that interfere with the binding of HIV to dendritic cell-specific intercellular adhesion molecule 3-grabbing non-integrin (DC-SIGN), a C-type lectin on human dendritic cells (DCs) [39,40,41,42,43]. All HMOs contain one lactose moiety attached to one or more N-acetyl-lactosamine or lacto-N-biose units, which are further attached to one or more units of either fucose or sialic acid, forming fucosylated and sialylated HMOs [35,36]. Lacto-N-fucopentaose III (LNFPIII) has an α1,3-linked fucose residue and exhibits immunomodulatory activity with no documented adverse effects. We have previously shown that, when conjugated to dextran, LNFPIII (P3DEX) activates CD14/TLR4 signaling for the extracellular signal-regulated kinase (ERK)-dependent production of anti-inflammatory mediators in pathological disease conditions such as diet-induced obesity, experimental autoimmune encephalomyelitis (EAE), and Gulf War Illness (GWI) [44,45,46,47,48]. However, the effect of P3DEX on viral infection-induced inflammation is unknown.

The purpose of the present study was to determine if P3DEX has anti-HIV and anti-inflammatory activity using primary human macrophages. We present evidence that P3DEX treatment can inhibit the intracellular replication of HIV without affecting its binding to and/or internalization of macrophages. We further show that P3DEX treatment inhibits proinflammatory cytokine gene expression irrespective of replicating or non-replicating HIV. These results provide evidence of the anti-HIV activity of P3DEX that can simultaneously control HIV-induced immune activation, suggesting its use as a host-directed therapeutic for people living with HIV (PWH).

## 2. Materials and Methods

Patient population: This study was reviewed and approved by the Institutional Review Board of the University of Georgia, Athens, GA, USA. The donors were healthy adults who were HIV-seronegative who provided written informed consent prior to donating blood.

Cell isolation and generation of primary human macrophages: Macrophages were prepared using the plate adherence method and characterized as previously described [49]. Peripheral blood mononuclear cells (PBMCs) were isolated from freshly obtained blood by Ficoll density centrifugation (GE Healthcare) to subsequently generate primary monocyte-derived macrophages (hereafter referred to as macrophages). For this, CD14+ monocytes were cultured with RPMI1640 supplemented with AB human serum and M-CSF (10 ng/mL) for 7 days, replacing M-CSF-supplemented fresh culture medium every third day.

Preparation of P3DEX conjugate: LNFPIII was synthesized by Dr. Peng George Wang (Georgia State University, Atlanta, GA, USA) [50,51]. LNFPIII (MW: 853.877 g/mol) was sent to Dr. Thomas Norberg (Uppsala University, Uppsala, Sweden) for conjugation to Food and Drug Administration (FDA)-approved aminodextran (DEX, 40 kDa average Mw, from Invitrogen, prod # D1861) using an APD linker-spacer method [52]. On average, conjugates had 10–12 LNFPIII monomers per 40 kDa dextran carrier, as determined by H-NMR spectroscopy. LNFPIII accounted for ~17–20% of the molecular weight of the P3DEX conjugate.

HIV-1 preparation and treatment: HIV-1BaL (HIV) was obtained through the NIH AIDS Research and Reference Reagent Program. Virus stocks were prepared following previously established procedures [53,54]. The Spearman–Karber method was used to determine the 50% tissue culture infectivity dose (TCID50) using the Alliance HIV-1 p24 antigen ELISA (Perkin Elmer Life Sciences). Cells were infected in antibiotic-free medium with the HIVBaL strain (TCID50 5.8) at a multiplicity of infection (MOI) of 0.01 for 5 h; subsequently, the cells were washed with phosphate-buffered saline (PBS) to remove extracellular viral particles and cultured in fresh medium. Cells were then treated with P3DEX or DEX at 6.25, 12.5, 25, and 50 µg/mL for 12 days. On day 3 and day 6, 250 µL of medium was removed from each well and replaced with fresh medium without any treatment or containing P3DEX or DEX. On day 12, all medium was collected from each well and stored at −80 °C until use. Virus binding was assessed by preincubating macrophages with CCR5 inhibitor Maraviroc (10 µM) or P3DEX (50 µg/mL) at 37 °C, under 5% CO_2_, for 1 h, followed by infection with HIV for 3 h. After 3 h, unbound virions were removed by extensively washing with Dulbecco’s phosphate-buffered saline, followed by the lysis of cells in 120 µL of CelLytic M (Sigma, St. Louis, MO, USA) supplemented with protease inhibitors (Thermo Scientific, Waltham, MA, USA). HIV content was assessed by monitoring p24 by ELISA [55]. Similar experimental conditions were used to estimate viral entry, with the exception that an additional step of trypsinization for 5 min at 37 °C was performed after 5 h to remove non-internalized virions [55]. The cells were then washed once with RPMI supplemented with 10% serum and three times with PBS before lysis.

Measurement of cell cytotoxicity: The cell cytotoxicity of macrophages infected with HIV and treated with P3DEX or DEX was determined using the CyQUANTTM LDH cytotoxicity assay kit, according to the manufacturer’s instructions [49]. Briefly, 50 µL of culture supernatant was added to an equal volume of reaction mixture and incubated at room temperature for 30 min, followed by the addition of a stop solution. Absorbance was measured at 490 nm and 680 nm as the reference wavelengths. Cells treated with lysis buffer and untreated media served as controls for maximum LDH and spontaneous LDH activity, respectively: % cytotoxicity = [Treatment LDH activity − spontaneous LDH activity/maximum LDH activity − spontaneous LDH activity] × 100. Data were presented as the percentage viability = 100 − % cytotoxicity.

Measurement of HIV: Replication was measured as the accumulation of HIV Gag p24 in culture supernatants [54]. The quantity of p24 in culture supernatants was measured using a Gag p24 ELISA kit (R&D systems). To determine the concentration of P3DEX needed to inhibit HIV replication by 50% (Inhibitory Concentration50—IC50), p24 was measured in 12-day-post-infection culture supernatants. Bound and internalized HIV was determined by measuring the quantity of p24 in HIV-infected macrophage lysates with or without treatment (internalized HIV and bound HIV, respectively).

Quantification of chemokines: The supernatants collected and stored at −80 °C were used to determine the levels of MIP-1α (CCL3), MIP-1β (CCL4), and CCL5 by ELISA (R&D systems), according to the manufacturer’s instructions.

Quantitative reverse transcriptase PCR (qRT-PCR): Total RNA was isolated from macrophages using TRIzolTM reagent, followed by treatment with DNase to remove DNA contamination (Thermo Fisher Scientific, Waltham, USA), according to the manufacturer’s protocol. RNA (500 ng) was used for cDNA synthesis using iScript advanced cDNA kit (Bio-Rad). The cDNA was stored at −20 °C until further analysis by qRT PCR. SyBrGreen master mix (Thermo Fisher Scientific) was used in a 10 µL reaction volume that included 10 ng of cDNA for primer pairs (1 µM) specific for respective human genes. Human HuPO was used as a housekeeping gene. The primer sequences used in this study, with their respective melting temperatures, are listed in Table 1. Data were analyzed to calculate the relative quantification of the gene of interest in comparison to the beta-actin gene by the comparative Ct method (2^−ΔCt^).

Immunoblotting: The immunoblotting of cellular lysates was performed as previously described [49,56]. The relative densities for target protein bands NFkB (65 kDa), pSer276NFkB (65 kDa), LC3B-II (14 kDa), and p62 (62 kDa) were normalized to housekeeping β-actin (45 kDa) or GAPDH (37 kDa) bands compared using ImageJ 1.52q (NIH). Normalized ratiometric data were log2 transformed.

Statistical analysis: Data were expressed as mean values ± standard error of the mean (SEM). Paired Student’s *t*-tests were used to determine the statistical significance for the in vitro experiments. IC50 of P3DEX was calculated using an inhibitor vs. response (three parameters) nonlinear regression curve with a 95% confidence interval (CI). Statistical analysis was performed using GraphPad Prism 10 (La Jolla, CA, USA). *p*-values of <0.05 were considered statistically significant.

## 3. Results

### 3.1. P3DEX Inhibits HIV-1 Replication in Human Macrophages

Macrophages are key target cells for HIV, playing key roles in acute and chronic phases of infection. During the acute phase, macrophages help establish infection at the site of viral entry, thus supporting HIV replication, which can be controlled by antiretroviral therapeutics [13,15,19,28,29,30]. Therefore, we sought to determine if P3DEX could inhibit the intracellular replication of HIV in primary human macrophages. For this, initially, we determined if primary human macrophages supported intracellular HIV replication in vitro. By measuring the accumulation of HIV Gag p24 in the culture supernatants as an indicator of HIV replication, similar to published studies, we found a progressive increase in the quantity of p24 over a 12-day infection period (Figure S1A), thus establishing the use of this model system to evaluate the anti-HIV activity of P3DEX.

**Figure 1:**
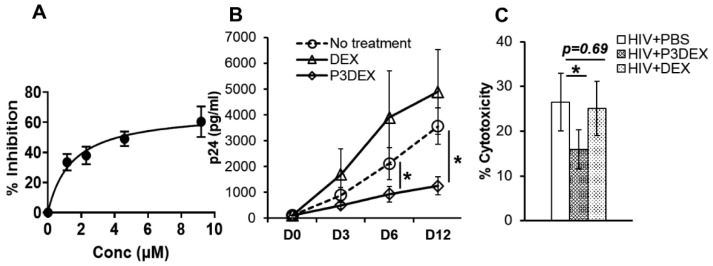
LNFPIII inhibits HIV replication in primary human macrophages: Macrophages were infected with HIV at MOI 0.01 for 5 h, followed by washing with PBS to remove unbound virions. Fresh medium was added. (**A**) Fresh medium was supplemented without or with 2-fold-increasing concentrations of P3DEX. The quantity of HIV Gag p24 was measured in the culture supernatant by ELISA on day 12 post infection. IC50 was calculated by nonlinear regression. (**B**,**C**) On day 3 and day 6, 250 µL of medium was removed from each well and replaced with fresh medium supplemented with P3DEX or DEX at 50 µg/mL. (**B**) The quantity of HIV Gag p24 was measured in the culture supernatant by ELISA at the indicated time points. (**C**) The quantity of lactate dehydrogenase was measured in the culture supernatant on day 12 post infection and presented as percentage viability = 100 − % cytotoxicity. Results are the mean values +/− SEM. The data shown are for (**A**) *n* = 4 donors and (**B**,**C**) *n* = 5 donors. * *p* < 0.05.

To test if P3DEX inhibited viral replication, 2-fold-increasing concentrations of P3DEX were added to HIV-infected macrophages. The quantity of p24 in the culture supernatants was measured 12 days post infection, and the concentration of P3DEX that could inhibit 50% of HIV activity (IC50) was calculated. We found that P3DEX had an IC50 of 1.5 µM and inhibited HIV replication in primary macrophages by almost 60% at 10 µM (50 µg/mL) (Figure 1A). Notably, the treatment of HIV-infected macrophages with DEX did not inhibit HIV replication at any examined concentration (Figure S1B).

We further determined the anti-HIV activity of 50 µg/mL P3DEX over a 12-day infection period. For this. the quantity of p24 in the culture supernatants was determined on days 0, 3, 6, and 12 post infection and compared with HIV-infected cells that had no treatment or had been treated with DEX. Compared to untreated macrophages, P3DEX treatment inhibited HIV replication as early as day 3 post infection (892.5 ± 288.9 vs. 497.73 ± 133.2 pg/mL; *p* = 0.1), with p24 levels significantly reduced on day 6 (2108.8 ± 614.9 vs. 935.12 ± 304.2 pg/mL; *p* = 0.03) and day 12 (3560.5 ± 708.8 vs. 1248.5 ± 351.3 pg/mL; *p* = 0.02) post infection (Figure 1B). By measuring the LDH in culture supernatants on day 12 post infection, we could determine whether the reduction in HIV was due to P3DEX-induced cytotoxicity. Compared to HIV-infected untreated macrophages, P3DEX treatment reduced HIV-induced % cytotoxicity (26.52 ± 6.4 vs. 15.88 ± 4.37%; *p* = 0.01) (Figure 1C). DEX treatment did not affect HIV-induced cytotoxicity (26.52 ± 6.4 vs. 25.06 ± 6.06%; *p* = 0.69). Collectively, these findings suggest that P3DEX can inhibit intracellular HIV replication without causing cell death.

### 3.2. P3DEX Does Not Inhibit HIV-1 Binding and Internalization

**Figure 2:**
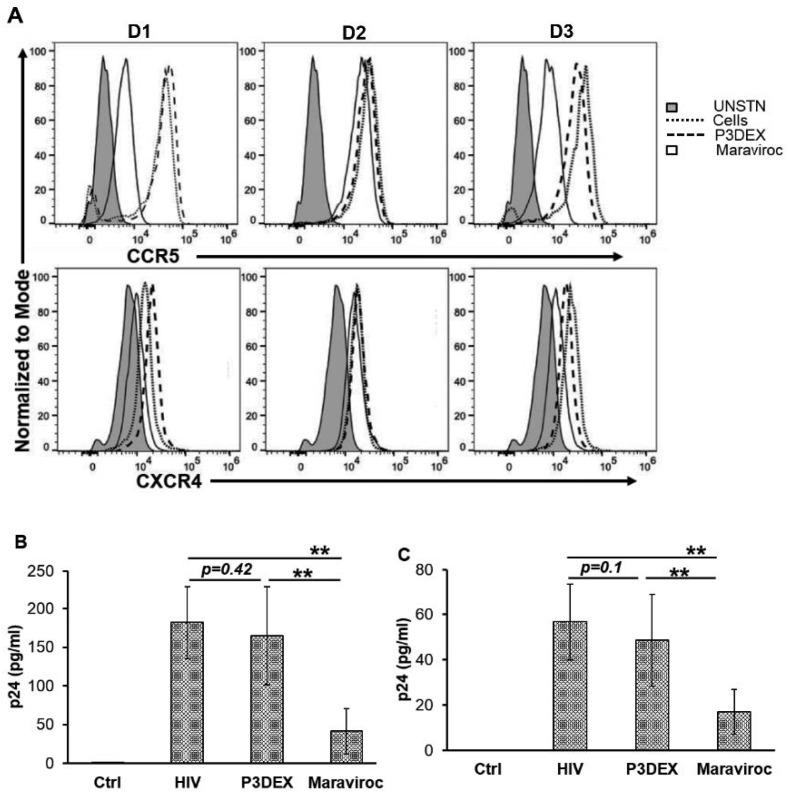
P3DEX does not inhibit HIV-1 binding and internalization. Macrophages were left untreated or preincubated with P3DEX (50 µg/mL) or CCR5 antagonist Maraviroc (5 µM) for 1 h. (**A**) Cells were surface-stained with anti-CCR5 and –CXCR4 antibodies, and their expression was measured by flow cytometry. (**B**,**C**) Cells were infected with HIV for 5 h, followed by the removal of unbound virions. (**B**) Cellular lysates were prepared. (**C**) Cells were trypsinized for 5 min at 37 °C to remove non-internalized virions, followed by cell lysate preparation. The quantity of HIV Gag p24 in the cellular lysates was measured by ELISA. The results are mean values +/− SEM. The data shown are for (**A**) *n* = 3 donors and (**B**,**C**) *n* = 4 donors. ** *p* < 0.005.

To understand how P3DEX affects HIV replication, we examined the sequential steps of HIV replication. P3DEX treatment did not significantly affect the expression of HIV receptors CCR5 or CXCR4, which are essential for HIV binding and entry into the host cells (Figure 2A). Consistent with this finding, the binding of HIV to P3DEX-treated cells, as measured by ELISA of cell-associated p24, was similar to that of untreated cells (165.2 ± 61 vs. 182.3 ± 47 pg/mL; *p* = 0.42) (Figure 2B). To determine the effect of P3DEX on virus entry, we examined the quantity of intracellular trypsin-resistant p24 associated with macrophages exposed to HIV for 5 h. Both untreated and P3DEX-treated cells displayed similar intracellular p24 concentrations over 5 h (56.85 ± 16.9 vs. 48.6 ± 20.5 pg/mL; *p* = 0.1) (Figure 2C). The treatment of cells with the CCR5 antagonist maraviroc was used as a positive control in these assays. Maraviroc significantly inhibited both the binding (182.3 ± 47 vs. 41.5 ± 29.2 pg/mL; *p* = 0.006) and internalization (56.85 ± 16.9 vs. 17 ± 0.0 pg/mL; *p* = 0.01) of HIV virions by the macrophages. Therefore, these experiments established that P3DEX inhibits HIV replication without affecting the early steps of HIV entry into macrophages.

### 3.3. P3DEX Increases β-Chemokine Production

Since P3DEX did not affect HIV binding and internalization but inhibited HIV viral replication, we wanted to further characterize the anti-viral activity. Chemokines MIP-1α (CCL3), MIP-1β (CCL4), and RANTES (CCL5) are produced as part of the immune response and demonstrate anti-HIV activity by competing with the virus for binding to the CCR5 receptor [57,58]. We sought to investigate the effect of P3DEX on β-chemokine production. For this, we measured the quantity of CCL3, CCL5, and CCL4 in the culture supernatants of macrophages infected with HIV and treated with P3DEX or DEX at 24, 48, and 72 h post infection.

**Figure 3:**
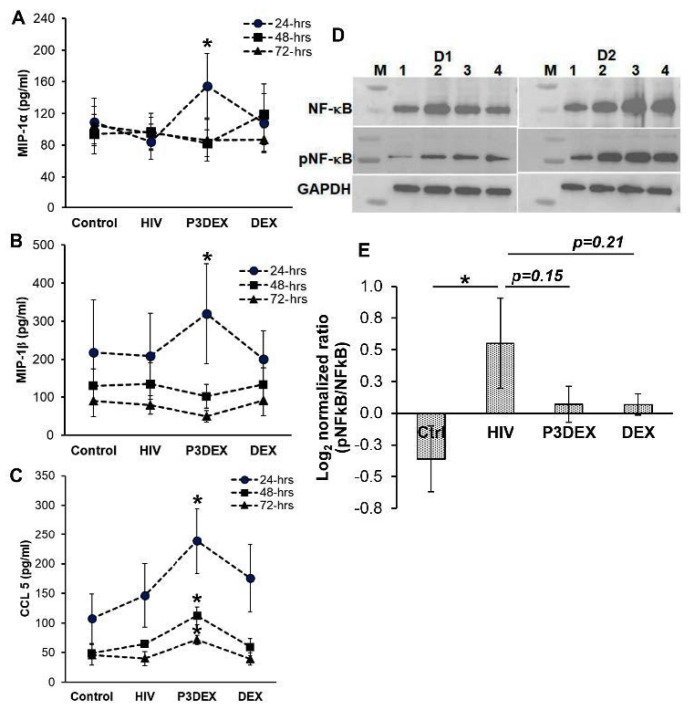
P3DEX increase β-chemokine production. Macrophages were infected with HIV_BaL_ at MOI 0.01 for 5 h, followed by washing with PBS to remove unbound virions. Fresh medium supplemented with P3DEX or DEX at 50 µg/mL was added. (**A**–**C**) The quantity of chemokines was measured in the culture supernatants by ELISA at the indicated time points. (**D**,**E**) Total cellular lysates were prepared and immunoblotted using anti-GAPDH (1:4000) or total NFκB p65 and -pNFκB p65 antibody (both at 1:1000). Immunoblots from lysates of 2 individual donors are shown; Lanes-M—molecular weight marker; 1—control; 2—HIV; 3—HIV + P3DEX; and 4—HIV + DEX (**D**). The results are mean values +/− SEM. The data shown are for (**A**–**C**) *n* = 5 donors and (**E**) *n* = 3 donors. * *p* < 0.05.

Compared to uninfected controls, HIV infection reduced the levels of CCL3 (MIP-1α) without affecting CCL4 (MIP-1β) and CCL5. The treatment of HIV-infected macrophages with P3DEX significantly increased the production of MIP-1α (84.48 ± 22 vs. 155 ± 40.4 pg/mL; *p* = 0.03), MIP-1β (208.14 ± 112 vs. 319.9 ± 131.2 pg/mL; *p* = 0.02), and CCL-5 (125.6 ± 26.54 vs. 198.53 ± 30.9 pg/mL; *p* = 0.02) 24 h post infection. The quantity of β-chemokines was comparable in HIV-infected–untreated and infected–P3DEX or DEX-treated macrophages (Figure 3A–C). By 48 and 72 h post infection, we observed that the levels of MIP-1α and MIP-1β chemokine production were similar between the untreated, DEX-, and P3DEX-treated groups. However, the quantity of CCL5 at 48 and 72 h post infection remained significantly elevated in HIV treated with P3DEX compared to untreated (70.16 ± 5.94 vs. 117.86 ± 12.7 pg/mL; *p* = 0.02 and 51.6 ± 11.9 vs. 72.94 ± 5.65 pg/mL; *p* = 0.03, respectively) or DEX-treated (72.24 ± 11.1 vs. 117.86 ± 12.7 pg/mL; *p* = 0.03 and 53.9 ± 10.7 vs. 72.94 ± 5.65 pg/mL; *p* = 0.02, respectively) HIV-infected macrophages. These results suggest that some of the anti-HIV activity of P3DEX is due to the upregulation of MIP-1α, MIP-1β, and CCL5 production. Further, the effect of P3DEX on CCL5 production was prolonged compared to other β-chemokines analyzed in this study.

β-chemokine signaling activates transcription factor NFκB, which regulates proinflammatory cytokine production. Concomitantly, HIV infection activates NFκB in a TLR8-dependent manner, which is important for the generation of full-length viral transcripts. Therefore, we sought to determine if P3DEX negatively impacted NFκB activation to control HIV replication. Compared to uninfected controls, HIV infection increased the phosphorylation of p65 subunit of NFκB (pNFκB) (−0.36 ± 0.26 vs. 0.55 ± 0.36; *p* = 0.01), while treatment with P3DEX did not affect pNFκB (0.55 ± 0.36 vs. 0.1 ± 0.14; *p* = 0.15) (Figure 3D,E). These findings suggest that P3DEX’s inhibition of HIV is independent of NFκB modulation.

### 3.4. P3DEX-Mediated Autophagy Inhibits HIV Replication

**Figure 4:**
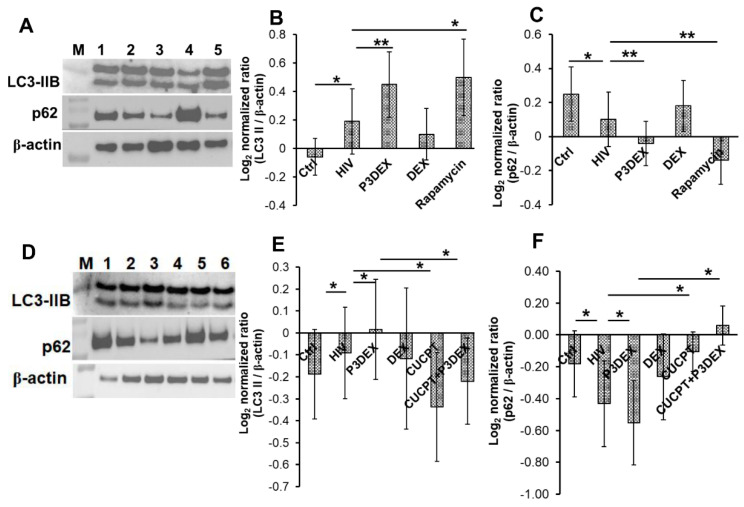
P3DEX augments autophagy in HIV-infected primary human macrophages. (**A**–**C**) Macrophages were infected with HIV at MOI 0.01 for 5 h, followed by washing with PBS to remove unbound virions. Fresh medium supplemented with P3DEX or DEX at 50 µg/mL or rapamycin (100 nM) was added. (**D**–**F**) Macrophages were left untreated or preincubated with TLR8 inhibitor CUCPT-9 (25 µM) for 45 min. Cells were infected with HIV for 5 h, followed by the removal of unbound virions. Fresh medium supplemented with P3DEX or DEX at 50 µg/mL or CUCPT-9 was added. Total cellular lysates were prepared and immunoblotted using anti-β-actin (1:4000), LC3B (1:1000), and p62 (1:5000) antibodies. Immunoblots from the lysates of representative donors are shown. Lanes-M—molecular weight marker; 1—control; 2—HIV; 3—HIV + P3DEX; and 4—HIV + DEX. Histograms are the mean values +/− SEM. The data shown are for (**B**,**C**) *n* = 5 donors and (**E**,**F**) *n* = 4 donors. * *p* < 0.05, ** *p* < 0.005.

Our findings so far suggest that P3DEX inhibits intracellular HIV replication without affecting NFκB-mediated HIV inhibition. Previous studies have demonstrated autophagy as an innate immune mechanism of macrophages to control HIV. Autophagy is a process where cytosolic microtubule-associated protein 1 light-chain 3B (LC3B)-I is converted to LC3B-II (LC3B lipidation). Increased LC3B-II expression, or its accumulation due to the inhibition of autophagic flux characterized by the degradation of polyubiquitin-binding protein p62 (sequestosome 1), is an indicator of autophagy induction. We studied autophagy in macrophages infected with HIV. Consistent with findings published by other research groups, we found that HIV infection marginally increased LC3B lipidation (−0.06 ± 0.13 vs. 0.19 ± 0.23; *p* = 0.05) and degraded p62 (0.25 ± 0.16 vs. 0.1 ± 0.16; *p* = 0.01). The treatment of HIV-infected macrophages with P3DEX substantially increased LC3B-II expression (0.19 ± 0.23 vs. 0.45 ± 0.23; *p* = 0.007) (Figure 4A,B) and p62 degradation (0.1 ± 0.16 vs. −0.04 ± 0.13; *p* = 0.004) (Figure 4A,C). Compared to HIV infection alone, DEX treatment had no effect on autophagy, and rapamycin, a known inducer of autophagy, significantly increased LC3B (0.19 ± 0.23 vs. 0.5 ± 0.27; *p* = 0.02) and decreased p62 levels (0.1 ± 0.16 vs. −0.14 ± 0.14; *p* = 0.005) (Figure 4A–C). Of note, treatment with P3DEX or rapamycin did not affect p62 at the transcription level and increased the expression of beclin, a key marker of autophagy (Figure S2A,B).

The autophagy-mediated control of HIV in macrophages occurs in a TLR8-dependent manner, so we sought to determine if P3DEX acted through the TLR8 pathway of autophagy. Initially, we treated HIV-infected macrophages with a 2-fold-increasing dose of TLR8 inhibitor CU-CPT9a and measured the quantity of IL-6 in the culture supernatants. We found that CU-CPT9a at 25 µM inhibits HIV-induced IL-6 production by 50% (Figure S3A). Subsequently, HIV-infected macrophages were treated with this suboptimal concentration of CU-CPT9a in the presence or absence of P3DEX, and autophagy was measured. Consistent with previous results, compared to infection alone, P3DEX treatment increased LC3B (−0.09 ± 0.2 vs. 0.02 ± 0.2; *p* = 0.05) (Figure 4D,E) and decreased p62 (−0.43 ± 0.27 vs. −0.55 ± 0.26; *p* = 0.02) expression (Figure 4D,F). However, suboptimal concentrations of CU-CPT9a inhibited autophagy (LC3B −0.09 ± 0.2 vs. −0.34 ± 0.2; *p* = 0.03, and p62 −0.43 ± 0.27 vs. −0.11 ± 0.12; *p* = 0.05) (Figure 4D–F). Of note, HIV-infected macrophages treated with P3DEX in the presence of CU-CPT9a, when compared to P3DEX alone, also inhibited autophagy, as observed by decreased LC3B (−0.22 ± 0.19 vs. 0.02 ± 0.2; *p* = 0.03) and increased p62 (0.06 ± 0.12 vs. −0.55 ± 0.26; *p* = 0.03) (Figure 4). Collectively, these findings suggest that P3DEX strengthens the anti-HIV activity of macrophages by augmenting autophagy in a TLR8-dependent manner.

**Figure 5:**
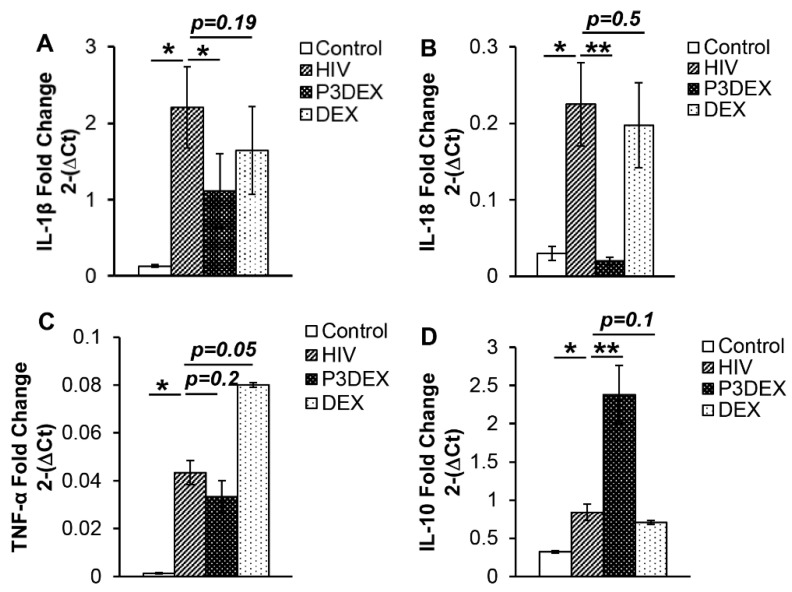
P3DEX inhibits proinflammatory cytokines’ expression. Macrophages were infected with HIV and treated with P3DEX or DEX at 50 µg/mL. Expression of housekeeping gene HuPO and cytokines genes (**A**) IL-1β, (**B**) IL-18, (**C**) TNFα, and (**D**) IL-10 was assessed using SYBR green, and fold-change was calculated. The results are mean values +/− SEM. The data shown are for (**A**) *n* = 5 donors, (**B**) *n* = 4, (**C**) *n* = 4, and (**D**) *n* = 5. * *p* < 0.05; ** *p* < 0.005.

Autophagy-mediated pathogen degradation limits inflammatory responses. In the following set of experiments, we sought to determine the effect of P3DEX on HIV-induced proinflammatory cytokines. We found that, compared to uninfected controls, HIV infection increased the expression of inflammatory cytokines IL-1β (0.13 ± 0.01 vs. 2.21 ± 0.6; *p* = 0.03), IL-18 (0.03 ± 0.01 vs. 0.23 ± 0.05; *p* = 0.03), and TNFα (0.001 ± 0.0002 vs. 0.04 ± 0.004; *p* = 0.03). However, treatment with P3DEX reduced the expression of IL-1β (2.21 ± 0.6 vs. 1.1 ± 0.4; *p* = 0.04) and IL-18 (0.23 ± 0.05 vs. 0.02 ± 0.005; *p* = 0.004) but did not affect TNFα (0.04 ± 0.004 vs. 0.03 ± 0.007; *p* = 0.23) expression (Figure 5A–C). DEX treatment did not affect proinflammatory cytokine expression. Concomitantly, we investigated the effect of P3DEX on the expression of the anti-inflammatory and regulatory cytokine IL-10. Compared to uninfected controls, HIV infection marginally increased the expression of IL-10 (0.32 ± 0.2 vs. 0.84 ± 0.25; *p* = 0.02). Treatment with P3DEX substantially increased IL-10 (0.84 ± 0.25 vs. 2.38 ± 0.41; *p* = 0.001). The IL-10 expression of HIV-infected macrophages treated with DEX was comparable to infected macrophages without any treatment (0.84 ± 0.25 vs. 0.71 ± 0.26; *p* = 0.1) (Figure 5D). Taken together, these studies suggest that P3DEX is an immune-regulatory HMO that augments the anti-HIV activity of macrophages by enhancing autophagy and controlling HIV-induced inflammation.

## 4. Discussion

The human milk oligosaccharide P3DEX has been effective in treating inflammation in chronic diseases [44,45,46,59,60]. Therefore, testing the effects of P3DEX on HIV infection as a way to better understand its therapeutic capabilities is pertinent not only to research related to HIV treatment but also to increase knowledge of the distinct effect of P3DEX on immune pathogenesis. In this study, we provide evidence that P3DEX inhibits intracellular HIV replication by augmenting autophagy-dependent HIV control mechanism of macrophages.

HMOs are a family of structurally diverse unconjugated glycans that are highly abundant in human milk [35,36,60,61]. HMOs serve as soluble decoys for viral, bacterial, or protozoan parasite adhesins, thereby preventing attachment to the infant mucosal surface. Secretory antibodies, tenascin-C, alpha-defensins, and long-chain polyunsaturated fatty acids can contribute to reduced transmission of HIV from mother to infant. Additionally, HIV-infected women with total HMOs above 1.87 g/L have been reported to be less likely to transmit HIV via breastfeeding [38,62,63,64,65]. Notably, the proportion of 3′-sialyllactose (3′-SL) is associated with increased HIV transmission and markers of more advanced disease in the mother, indicating that the specific profile of HMOs is important in determining the risk of transmission [38,66]. Oligosaccharide analysis of breastmilk samples collected from HIV-infected mothers in Lusaka, Zambia, determined that higher maternal breastmilk concentrations of 2-linked fucosylated HMOs (2′-fucosyllactose and lacto-N-fucopentaose I) and non-2-linked fucosylated HMOs (3-fucosyllactose and LNFP II/III) were associated with reduced mortality during breastfeeding in HIV-exposed but uninfected children [38,67]. Further clinical and humanized mouse model studies established that HMOs protect postnatal HIV transmission, inhibit the in vitro transfer of HIV to CD4+ T lymphocytes, and prevent HIV transmission in vivo [68]. The HMOs of mothers with HIV have higher relative abundances of 3′-sialyllactose compared to HIV-uninfected counterparts. Our findings provide the first evidence that LNFPIII exhibits direct HIV-inhibitory activity by augmenting an autophagy-mediated anti-viral mechanism. Bode et al. [38] found a nonsignificant trend toward a higher concentration of another oligosaccharide, lacto-N-neotetraose (LNnT), most abundantly present in human breast milk and a reduced risk of HIV (adjusted OR: 0.49; 95% CI: 0.23, 1.04). On testing the anti-HIV activity of LNnT, we found that the treatment of HIV-infected macrophages with LNnT neither inhibited intracellular HIV replication nor reduced proinflammatory cytokines gene expression, corroborating the findings of Bode et al. Meanwhile, Ramadhin et al. [59] previously compared P3DEX- and dextran-conjugated LNnT (NTDEX) in a mouse model of diet-induced obesity and also established that P3DEX ameliorated inflammation and hepatic lipid accumulation whereas NTDEX did not. These findings collectively suggest that slight structural differences alter therapeutic abilities and exemplify the differential roles that individual HMOs might execute.

β-chemokines are traditionally known to control HIV due to their competitive binding to HIV coreceptors. However, evidence shows that cells expressing mutant CCR5 or CRISPR-Cas9-mediated CCR5/CXCR4 gene deletion still support HIV entry, which is suggestive of the role of other coreceptors in viral entry and subsequent replication events [57,69,70]. We found that the P3DEX treatment of HIV-infected macrophages did not affect the surface expression of CCR5 or CXCR4. HIV infection is associated with a cytokine storm due to cellular activation as a result of phosphorylation and nuclear translocation of transcription factor NFκB (pNFκB). pNFκB binds to the promoter region of various cellular genes and the HIV long-terminal repeats (LTRs). The binding of NFκB to HIV LTR potentiates viral transcription, and this is further increased by TNFα and IL-1β cytokines in a TLR8-dependent manner [71,72,73,74,75]. Specifically, the p65 subunit of NFκB phosphorylated at Ser276 recruits transcription-elongation factor pTEF-b to the LTR, resulting in the phosphorylation of RNA polymerase II (RNAPII), critical for elongation and generation of full-length HIV transcripts [71,72,73]. Similarly to other reported studies, HIV infection in our study also increased pNFκB and proinflammatory cytokine (TNFα, IL-1β, and IL-18) gene expression in primary human macrophages. However, the P3DEX treatment did not affect pNFκB or TNFα but significantly reduced IL-1β and IL-18 and increased IL-10 expression. Notably, treatment with P3DEX reduced IL-1β and IL-18 in macrophages cultured with inactive HIV (unpublished findings). This suggests that P3DEX may be suitable for controlling immune activation (IA), which is prevalent in patients with ART-mediated viral suppression. In this study, we demonstrated the direct anti-HIV activity of P3DEX.

Our results, showing that autophagy is an innate defense mechanism of macrophages to control HIV, is consistent with previous works. Kinetic studies have shown a maximum increase in LC3B lipidation accompanied by p62 degradation in HIV-infected macrophages 24 h post infection. The p62 protein levels increased from day 3 through day 5, and by day 7, they were similar to uninfected macrophages. This corresponded to LC3B lipidation, which peaked 24 h post infection and continued to decline over 10 days [76]. Our findings here show that P3DEX potentiates autophagy in HIV-infected macrophages. Although we did not measure autophagy beyond 24 h of HIV infection and P3DEX treatment, the inhibition in intracellular HIV replication over 12 days, as observed in P3DEX-treated cells, was likely due to the continued activation of the autophagy-mediated phagolysomal pathway, leading to the inhibition of viral replication. Beyond the scope of this current study, it will be worthwhile to investigate the effect of P3DEX on HIV’s negative regulatory factor (Nef)-mediated inhibition of the proteolytic stages of autophagy to overcome the phagosome maturation block critical to the inhibition of viral replication [76,77,78]. Previous reports suggest that autophagy is a negative regulator of inflammasome activity by scavenging endogenous inflammasome activators, such as reactive oxygen superoxide (ROS), producing damaged mitochondria and reducing the accumulation of pro-IL1β [79,80]. Our findings suggest that P3DEX-induced autophagy also limits persistent immune activation by controlling inflammasome activation.

Lewis X, which is a partial structure of LNFPIII and some other human milk oligosaccharides, binds DC-SIGN to compete with HIV’s envelope protein gp120 for binding to DC-SIGN and inhibits HIV transfer to CD4+ T lymphocytes [39,40,41]. Srivastava et al. [47] showed that SIGNR-1, a mouse analog of human DC-SIGN, is one of the receptors for P3DEX. However, downregulating the expression of SIGNR-1 did not affect the uptake of P3DEX. Here, we showed that the P3DEX treatment of primary human macrophages did not affect the expression of HIV coreceptor CCR5 and CXCR4, or DC-SIGN (Figure S3B), consequently not affecting HIV binding or internalization. Notably, P3DEX directly inhibited intracellular HIV replication by augmenting autophagy to levels comparable to rapamycin. Rapamycin is an immune-suppressive macrolide with established anti-HIV activity through the inhibition of mTOR. Although rapamycin-based therapy has been used in patients with various types of solid tumors, its use is limited due to poor solubility, pharmacokinetics, and mild-to-serious and possibly life-threatening side effects, especially at higher doses [81,82,83,84]. Therefore, repurposing rapamycin for the clinical care of people living with HIV is yet to be evaluated in clinical settings. Given that HMOs are naturally occurring biomolecules present in abundance in human breast milk and already registered for use in infant formula in Europe (Novel Food Application 157) and the United States (GRAS Notice 659), they are a promising anti-HIV host-directed therapeutic that can be used now [85]. Specifically, the pre-clinical assessment of P3DEX in various disease models ameliorated disease outcomes with no adverse effects up to 75 µg weekly doses for 9 months in mice [44,45,59]. Here, we provided the first evidence of the HIV-inhibitory activity of P3DEX with no cytotoxicity to adult macrophages. Our findings on HIV-infected adult macrophages suggest that, despite the comparable concentration and relative abundance of P3DEX in the milk of lactating mothers with and without HIV, P3DEX supplementation can effectively control HIV across the lifespan.

## 5. Conclusions

In summary, our findings are the first demonstration of the inhibitory activity of P3DEX in adult macrophages to simultaneously control HIV and HIV-associated inflammation. We propose that P3DEX potentiates the inflammasome–autophagy axis to simultaneously control inflammation/IA and HIV replication.

## Figures and Tables

**Table 1:** Primers, with their respective annealing temperatures, used for RT PCR.

Gene	Primer Sequence	Temperature (°C)
*HuPO*	5′-CAT TCT ATC ATC AAC GGG TAC AA-3′ 5′-AGC AAG TGG GAA GGT GTA ATC C-3′	60
*SQSTM1*	5′-TAG GAA CCC GCT ACA AGT GC-3′ 5′-GAG AAG CCC TCA GAC AGG TG-3′	60
*BECN1*	5′-GAA GAC ACA GGA GGC AGT GG-3′ 5′-AGG ACA CCC AAG CAA GAC C-3′	60
*IL-1β*	5′-CTG GAC CTC TGC CCT CTG G-3′ 5′-TCC ATG GCC ACA ACA ACT GA-3′	60
*IL-18*	5′-GAT AGC CAG CCT AGA GGT ATG G-3′ 5′-CCT TGA TGT TAT CAG GAG GAT TCA-3′	57
*TNF-α*	5′-GCC CAG GCA GTC AGA TCA TC-3′ 5′-CGG TTC AGC CAC TGG AGC T-3′	60
*IL-10*	5′-TCT CCG AGA TGC CTT CAG CAG A-3′ 5′-TCA GAC AAG GCT TGG CAA CCC A-3′	58

## Data Availability

The original contributions presented in this study are included in the article/Supplementary Materials; further inquiries can be directed to the corresponding author.

## Supplementary Materials

The following supporting information can be downloaded at https://www.mdpi.com/article/10.3390/nu17050890/s1: Figure S1: HIV replication in primary human macrophages; Figure S2: P3DEX augments autophagy in HIV-infected primary human macrophages; and Figure S3: Effect of CUCPT-9 and P3DEX on primary human macrophages.
