# Supplementary material for: The Human Milk Oligosaccharide Lacto-N-Fucopentaose III Conjugated to Dextran Inhibits HIV Replication in Primary Human Macrophages
Source: Nutrients. 2025 Mar 2;17(5):890. doi: 10.3390/nu17050890 (PMC11901455; doi:10.3390/nu17050890)
Supplement: Supplementary file 1 [file nutrients-17-00890-s001.zip › nutrients-3343986-supplementary.pdf]

Supplementary Figure S1

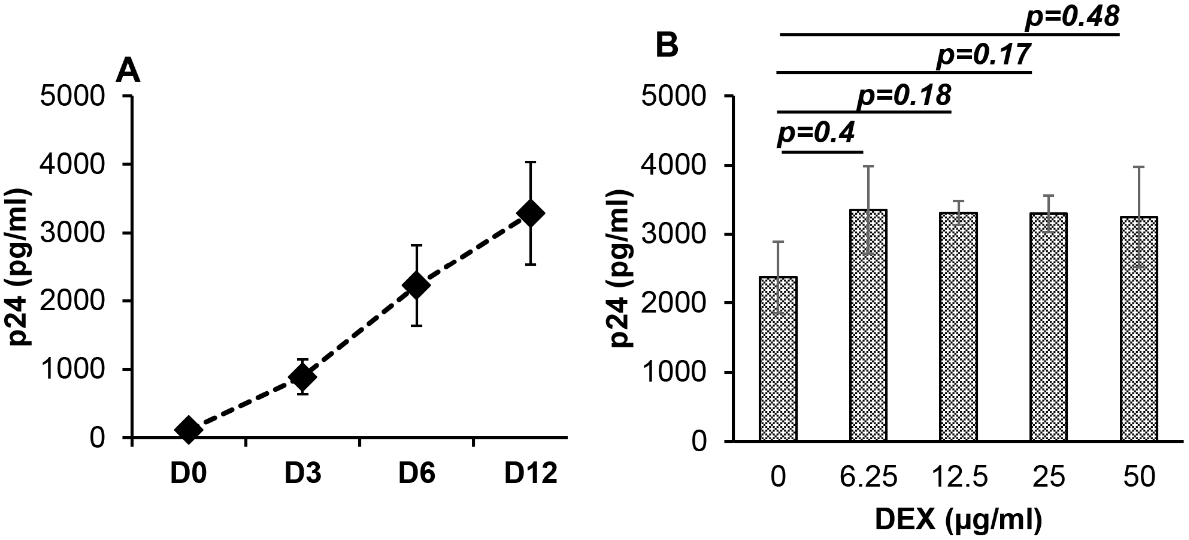

HIV replication in primary human macrophages: Macrophages were infected with HIV at MOI 0.01 for 5 hours followed by washing with PBS to remove unbound virions. Fresh medium was added. **(a)** The quantity of HIV Gag p24 was measured in the culture supernatants at the indicated time days (D) post-infection. **(b)** Fresh medium supplemented without or with a 2-fold increasing concentration of P3DEX**.** The quantity of HIV Gag p24 was measured in the culture supernatant by ELISA on day 12 post-infection. Results are Mean values +/- SEM. Data shown are for (a) N=8 donors, (b) N=4.

Supplementary Figure S2

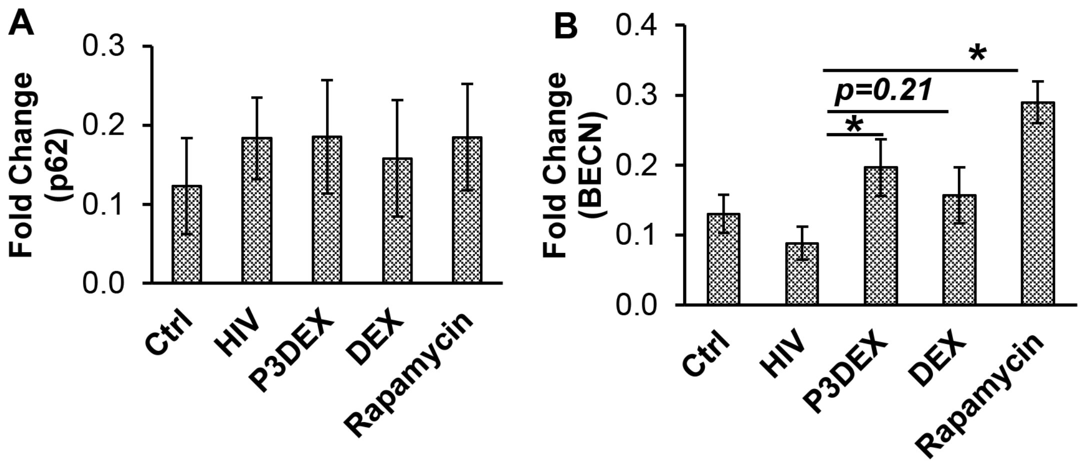

P3DEX augments autophagy in HIV-infected primary human macrophages: Macrophages were infected with HIV at MOI 0.01 for 5 hours followed by washing with PBS to remove unbound virions. Fresh medium supplemented with P3DEX or DEX at 50 µg/ml, or rapamycin at100 nM was added. Expression of housekeeping gene HuPO and autophagy genes (A) SQSTM (p62), and (B) Beclin-1 (BECN) was determined using SYBR green, and fold change was calculated. Results are Mean values +/- SEM. Data shown is for N=5 donors. *p<0.05

Supplementary Figure S3

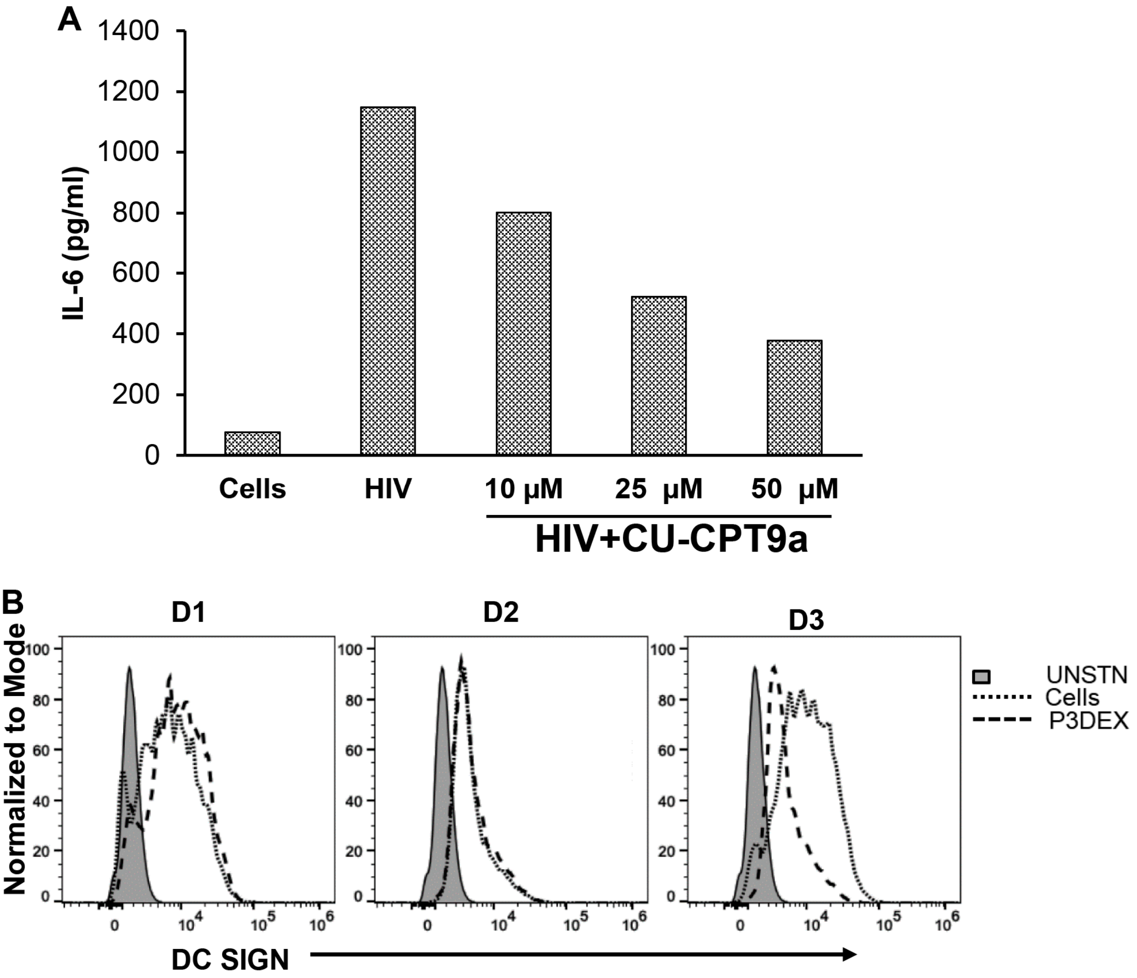

Effect of CUCPT-9 and P3DEX on primary human macrophages: (**a**) Macrophages were left untreated or preincubated with TLR8 inhibitor CUCPT-9 at the indicated dose for 45 minutes. Cells were infected with HIV infection for 5 hours followed by removal of unbound virions. Fresh medium supplemented with CUCPT-9 was added. The quantity of IL-6 was measured in the culture supernatants by ELISA. Results are Mean values of N=2 donors. (**b**) Macrophages were left untreated or preincubated with P3DEX (50 µg/ml) for 60 minutes. Cells were surface stained with anti-DC SIGN antibody and its expression was measured by flow cytometry. Data shown is from 3 donors.
